# Androgen receptor suppresses vasculogenic mimicry in hepatocellular carcinoma via *circRNA7/miRNA7‐5p/VE‐cadherin/Notch4* signalling

**DOI:** 10.1111/jcmm.16022

**Published:** 2020-10-28

**Authors:** Shixiang Bao, Shuai Jin, Chunhua Wang, Peipei Tu, Kongwang Hu, Jingtao Lu

**Affiliations:** ^1^ School of Life Sciences Anhui Medical University Hefei China; ^2^ Departments of General Surgery the First Affiliated Hospital of Anhui Medical University Hefei China; ^3^ Departments of Pathology and Urology University of Rochester Medical Center Rochester NY USA

**Keywords:** androgen receptor, circRNA7, hepatocellular carcinoma, miRNA7‐5p, Notch4, vasculogenic mimicry, VE‐Cadherin

## Abstract

Androgen receptor (AR) can suppress hepatocellular carcinoma (HCC) invasion and metastasis at an advanced stage. Vasculogenic mimicry (VM), a new vascularization pattern by which tumour tissues nourish themselves, is correlated with tumour progression and metastasis. Here, we investigated the effect of AR on the formation of VM and its mechanism in HCC. The results suggested that AR could down‐regulate circular *RNA (circRNA) 7*, up‐regulate micro *RNA (miRNA) 7‐5p*, and suppress the formation of VM in HCC *Small hairpin circR7 (ShcircR7)* could reverse the impact on VM and expression of *VE‐cadherin* and *Notch4* increased by small interfering AR (shAR) in HCC, while inhibition of *miR‐7‐5p* blocked the formation of VM and expression of *VE‐cadherin* and *Notch4* decreased by AR overexpression (oeAR) in HCC. Mechanism dissection demonstrated that AR could directly target the *circR7* host gene promoter to suppress *circR7*, and *miR‐7‐5p* might directly target the *VE‐cadherin* and *Notch4* 3′UTR to suppress their expression in HCC. In addition, knockdown of *Notch4* and/or *VE‐cadherin* revealed that *shVE‐cadherin* or *shNotch4* alone could partially reverse the formation of HCC VM, while *shVE‐cadherin* and *shNotch4* together could completely suppress the formation of HCC VM. Those results indicate that AR could suppress the formation of HCC VM by down‐regulating *circRNA7/miRNA7‐5p/VE‐Cadherin/Notch4* signals in HCC, which will help in the design of novel therapies against HCC.

## 1 INTRODUCTION

Hepatocellular carcinoma (HCC), which accounts for more than 90% of liver cancers, is ranked as the fourth most common malignancy.^1^ It is also the fourth leading cause of cancer‐induced death, with more than 780 000 deaths every year.^2^ While molecular targeting chemotherapy and surgical resection have recently experienced rapid progress, the advancement of long‐term survival remains far from satisfactory. Moreover, patients receiving surgical resection often have a high rate of recurrence, with a five‐year survival only in near 25% of cases.^3^, ^4^ Currently, detailed mechanisms for HCC initiation and progression remain unclear.

Androgen receptor (AR) is a nuclear receptor that plays a key role in influencing HCC progression.^5^ Some reports have indicated that AR can promote HCC development at earlier stages,^6^, ^7^, ^8^, ^9^ and that inhibition of AR suppressed hepatitis B virus (HBV)‐ or carcinogen‐induced HCC progression from early stages in vitro and in vivo.^8^ Inhibiting AR can increase HCC metastasis in advanced stages; therefore, AR likely inhibits HCC metastasis in advanced stages. This might account for the conflicted outcomes of clinical trials using different anti‐androgens to treat HCC.^9^ Ma et al reported that AR could suppress HCC metastasis in HCC cell lines and in advanced stage HCC in L‐ARKO mice. AR together with Sorafenib might suppress HCC late‐stage progression.^10^ However, the molecular mechanisms underlying how activated AR signals suppress HCC late‐stage progression remain to be elucidated.

Vascularization plays a crucial role in tumour metastasis.^11^ The progression of cancer depends on tumour vascularization, as new vessel formation ensures a sufficient supply of oxygen, nutrients and growth factors to the growing tumour and facilitates tumour dissemination. Recent studies have revealed a vasculogenic mimicry (VM) pattern, a new pattern where tumour tissues nourish themselves. The pattern includes vessels lined exclusively with tumour cells mimicking the presence and function of endothelial cells, as one means for blood supply to tumours.^12^, ^13^ VM has been identified in many aggressive metastatic tumours, showing that some tumour cells are able to form highly patterned vascular channels in vitro. These channels consist of a basement membrane that stains positive with the VM marker periodic acid‐Schiff (PAS) in the absence of CD31‐positive endothelial cells.^14^ Maniotis et al^15^ reported that tumour cells, rather than endothelial cells, can form blood vessels in highly aggressive uveal melanomas, and the phenomenon of tumour vascularization was described as vascular mimicry. As that early description, VM has been found in prostatic carcinoma,^16^ breast carcinoma,^17^ ovarian carcinoma,^18^ lung cancer^19^ and pancreatic carcinoma.^20^ Recent studies have indicated that VM is also associated with metastasis and invasion, and is implicated in poor prognosis of hepatocellular carcinoma^21^ and gastrointestinal stromal tumours.^22^ However, whether AR can influence VM formation in HCC is still unclear. In this study, we investigated the effect of AR on the formation of VM and its mechanism in HCC.

## 2 MATERIALS AND METHODS

### 2.1 Materials

AR, VE‐cadherin and GAPDH antibodies for Western blotting were purchased from Santa Cruz Biotechnology. Notch4 antibody for Western blotting analysis was purchased from Biorbyt. Anti‐mouse/rabbit secondary antibodies for Western blotting were from Invitrogen.

### 2.2 In vitro cell culture/maintenance

The SKhep1 human HCC cell line was purchased from the American Type Culture Collection (ATCC). The HA22T human HCC cell line was purchased from the Food Industry Research and Development Institute in Taiwan (BCRC number: 60168). All cell lines were cultured in DMEM (Invitrogen), supplemented with 10% foetal bovine serum (v/v), penicillin (25 U/mL, streptomycin (25 g/mL) and 1% L‐glutamine. All cell lines were cultured in an atmosphere containing 5% (v/v) CO_2_ at 37°C.

### 2.3 VM formation assay and quantification

SK and HA22T cells that overexpressed AR or were transfected with knockdown vectors were trypsinized, then resuspended in serum‐free DMEM at a concentration of about 5 × 10^5^/mL. Human microvascular endothelial cells were cultured and used as a positive control. A total of 50 µL of Matrigel (BD Bioscience) were plated in 96‐well plates and incubated at 37°C for 1 hour. Then, 100 µL of resuspended SK or HA22T cells was loaded on the Matrigel. Three replicate wells were used for each condition. Following incubation for 4‐6 hours at 37°C, 100 µL of 10% formalin was gently added to each well for 10 minutes to fix VM. Each well was analysed under a microscope with 10× phase contrast. VM numbers in each field were imaged and 3‐5 fields in each well were randomly selected to calculate the average value.

### 2.4 RNA extraction, miRNA extraction, reverse transcription and quantitative real‐time PCR analysis

Total RNA was extracted from HCC cells using Trizol reagent (Invitrogen). A total of 1 µg of extracted RNA were reverse transcribed into cDNA using Superscript III transcriptase (Invitrogen). Quantitative real‐time PCR (qRT‐PCR) was used to determine the mRNA expression levels of target genes. Expression levels were normalized to the expression of *GAPDH* RNA.

miRNAs were extracted using the PureLink® miRNA kit. Briefly, 2 µg of RNA was processed for poly A polymerase addition by adding 1 mmol/L ATP and 1 U of polymerase in 1x RT buffer for 15 minutes at 37°C in a total of 10 µL. Reverse transcription was performed by annealing at 65°C for 5 minutes, followed by 4°C for 2 minutes after adding 50 µm of the RT anchor primer. Finally, cDNA was synthesized at 42°C for 1 hour after adding 2 µL 10 mmol/L dNTP, 2 µL 5x RT buffer, 1 µL reverse transcriptase and ddH_2_O to a total 20 µL. QRT‐PCR was used to determine miRNA expression levels. Expression levels were normalized to the expression of 5s RNA and/or U6.

### 2.5 Western blot analysis

Cells were harvested and lysed in lysis buffer. Protein concentrations were measured, followed by heating under reducing conditions. A total of 30 µg of protein were loaded on SDS‐PAGE gels and then transferred onto PVDF membranes. After blocking, they were blotted with the following antibodies: GAPDH, AR, Notch4 and VE‐cadherin.

### 2.6 Lentiviral‐based gene delivery

293T cells were transfected with pWPI‐AR, PLV‐THM plasmid, psAX2 packaging plasmid, and pMD2G envelope plasmid to generate lentivirus expressing AR. The lentivirus was collected and frozen at −80°C for later use.

### 2.7 Luciferase assay

Fragments (540 bp) of *VE‐cadherin* and *Notch4* 3′UTR containing mutant or wild‐type miRNA‐responsive elements were cloned into the psiCheck2 construct (Promega) downstream of the Renilla luciferase ORF. Fragments (2600 bp) of *circRNA7* promoter containing mutant or wild‐type miRNA‐responsive elements were cloned into the pGL3 construct (Promega) downstream of the Renilla luciferase ORF. SK cells were plated in 24‐well plates for 24 hours; then, the DNA was transfected into SK cells with Lipofectamine 3000 (Invitrogen) according to the manufacturer's instructions. Luciferase activity was assayed by dual‐luciferase assay (Promega), according to the manufacturer's protocol.

### 2.8 Chromatin immunoprecipitation assay (ChIP)

SK cells were crosslinked with 1% formaldehyde for 10 minutes, followed by quenching with glycine. Sonication was performed to obtain genomic DNA fragments of approximately 200‐400 bp. Sonicated DNA was immunoprecipitated with 2.0 µg anti‐AR antibody (Santa Cruz Biotechnology) at 4°C overnight. IgG was used in the reaction as a negative control. A target sequence within the human *circRNA7* promoter was detected by PCR with specific primes (Table S1).

### 2.9 Statistical analysis

Quantitative data were expressed as mean ± standard deviation (SD) from at least three independent experiments. Statistical analyses were performed using the Student's *t* test with GraphPad Prism 10 (GraphPad software, Inc). Statistical significance among the control and treated groups was analysed by ANOVA, and *P < .05 was regarded as the threshold value for statistical significance.

## 3 RESULTS

### 3.1 AR suppresses VM formation in SK and HA22T cells

**FIGURE 1:**
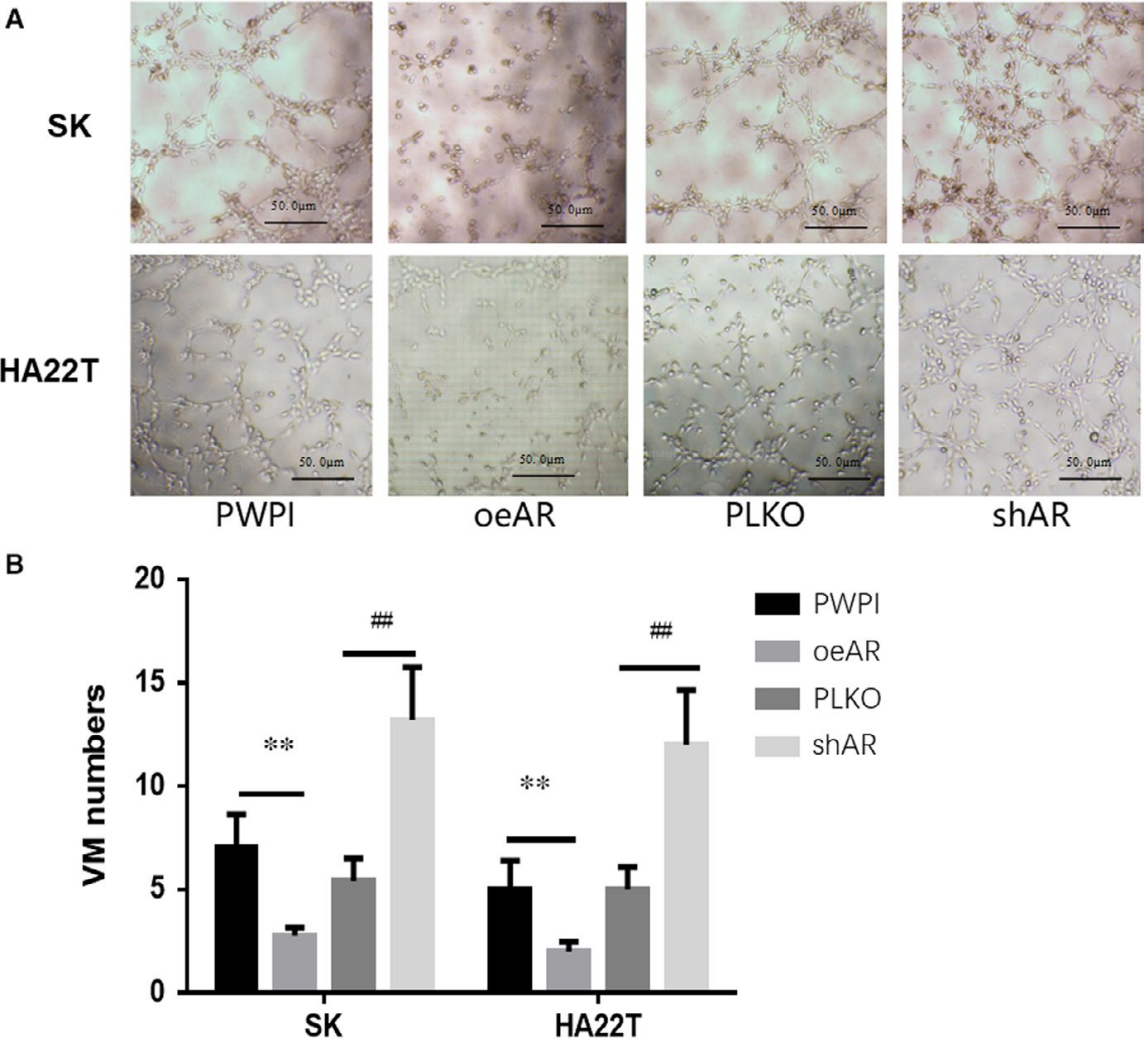
AR suppresses VM formation in SK and HA22T cells. (A) AR suppresses VM formation in SK cells. (B) AR suppresses VM formation in HA22T cells. (C) Quantification of the formation of VM in SK and HA22T cells. The number of VM formations was counted and compared. **P < .01, compared with the PWPI control group; ^##^ *P* < .01, compared with the PLKO control group

Recent studies indicated that AR might suppress HCC metastasis in vitro and in vivo, and might particularly suppress HCC late‐stage progression. VM is a type of vascularization which ensures an adequate supply of oxygen, nutrients and growth factors to the growing tumour and facilitates tumour dissemination. It has also been shown to contribute to tumour metastasis. To test whether AR suppresses HCC metastasis through regulating VM formation, we manipulated AR expression in the VM formation process. We found that overexpression of AR (oeAR) could suppress significantly HCC VM formation in both SK and HA22T cells. Consistent with this finding, reducing AR expression with *shAR* in HCC cells significantly increased VM formation (Figure 1).

### 3.2 AR suppresses the expression of *VE‐cadherin* and *Notch4* in HCC

**FIGURE 2:**
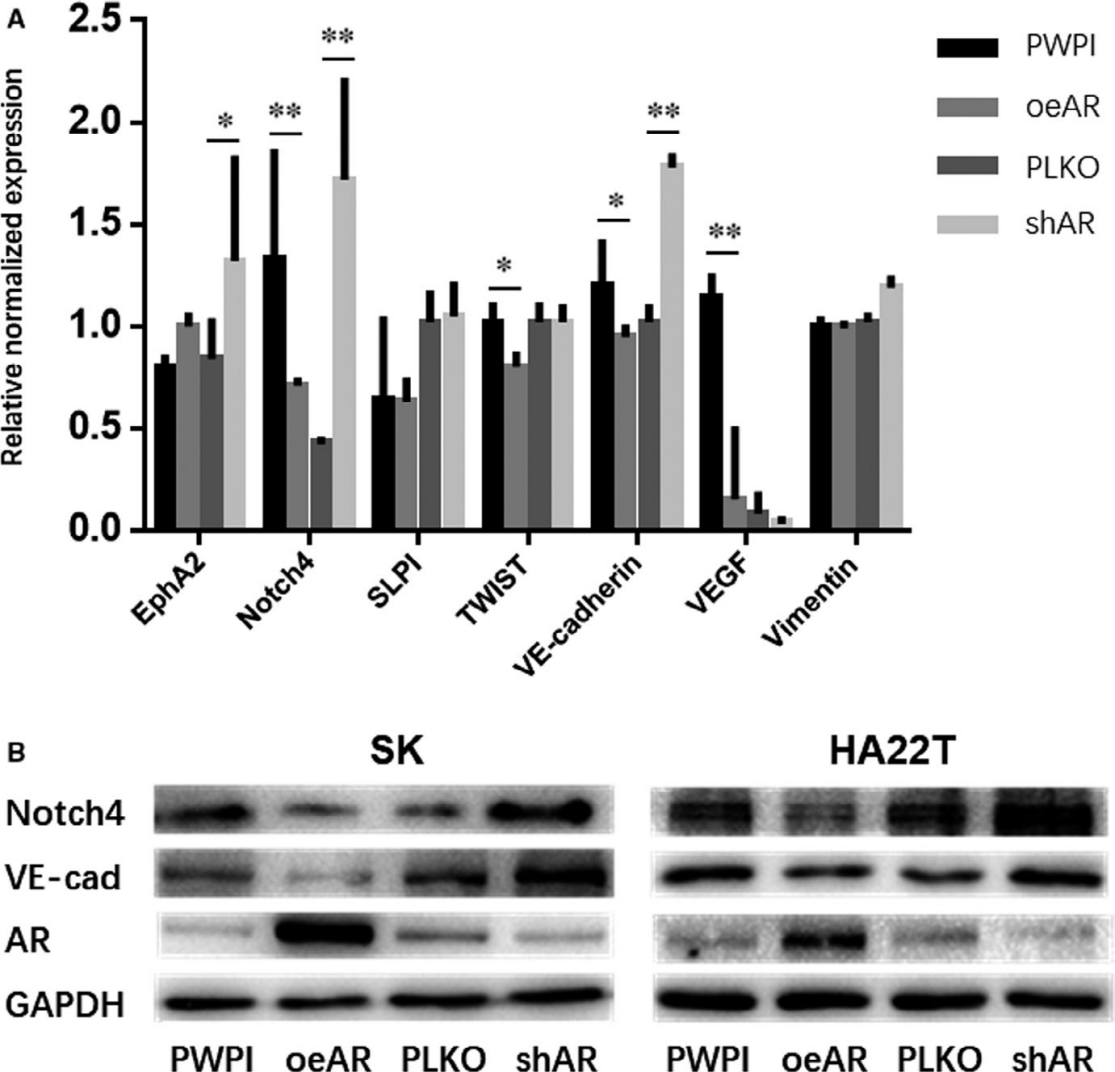
AR suppresses the expression of *VE‐cadherin* and *Notch4* in HCC. (A) The effect of AR on gene expression of *EphA2, Notch4, VE‐Cadherin, SLPI, TWIST, VEGF* and *vimentin* in SK cells. (B) The effect of AR on protein expression of AR, VE‐cadherin and Notch4. qRT‐PCR and Western blotting were performed as described in the Materials and Methods. *P < .05, **P < .01, compared with control group

Many factors, including EphA2, Notch4, VE‐cadherin, SLPI, TWIST, VEGF, vimentin and HIF‐1α, are related to the formation of VM in many cancers. Recent studies indicated that Notch4, VE‐Cadherin, TWIST and VEGF participated in the formation of VM in HCC.^23^, ^24^, ^25^ Therefore, we focused on the potential role of AR in regulating the expression of VM‐related factors in HCC cells. Through qRT‐PCR, we found that oeAR inhibited gene expression of *Notch4* and *VE‐cadherin*, while *shAR* increased the expression of both genes in SK cells (Figure 2A). To further confirm the effect of AR on the expression of VE‐cadherin and Notch4 in SK and HA22T cells, we determined their protein levels under the same conditions. Similar to changes in mRNA expression, oeAR inhibited protein expression of Notch4 and VE‐cadherin, while *shAR* increased expression of both proteins in SK and HA22T cells (Figure 2B). These results suggest that AR can suppress the formation of HCC VM by inhibiting the expression of Notch4 and VE‐cadherin.

### 3.3 AR suppresses the formation of HCC VM by inhibiting expression of *Notch4* and *VE‐cadherin*

**FIGURE 3:**
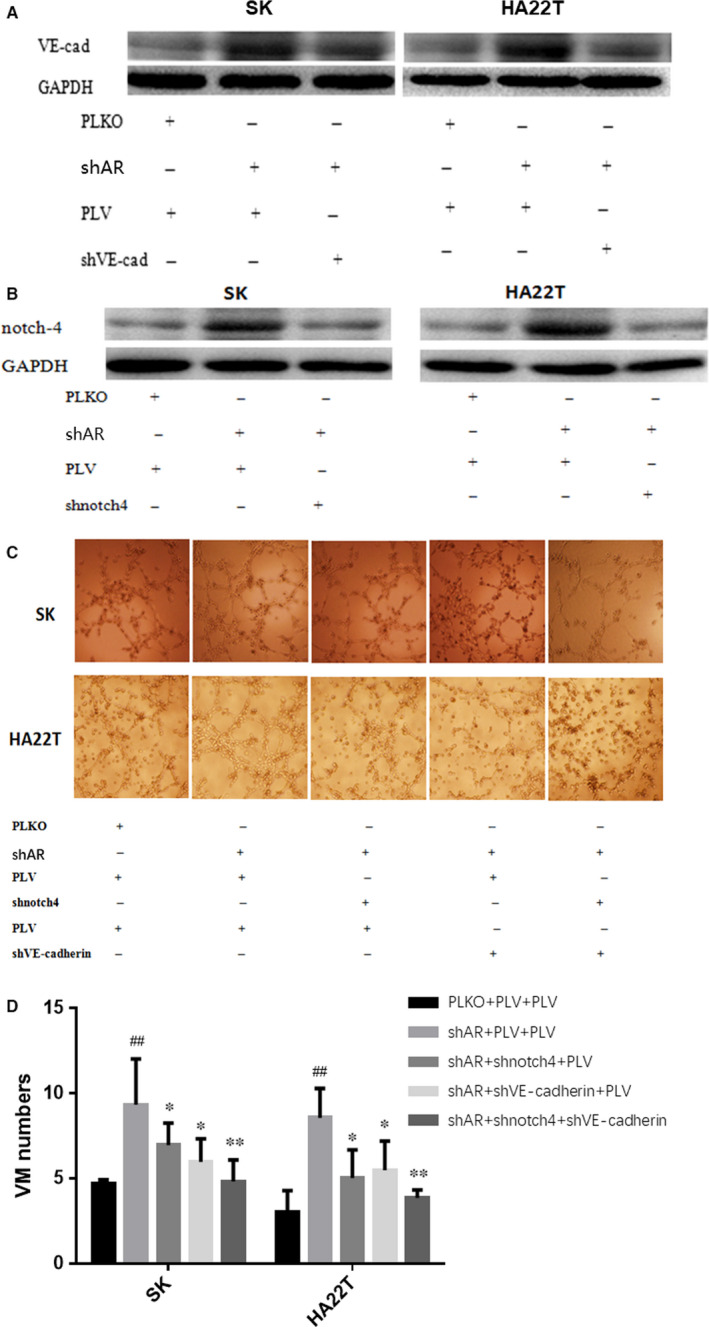
AR suppresses the formation of HCC VM by inhibiting expression of *Notch4* and *VE‐cadherin*. (A) *shVE‐cadherin* inhibits the expression of *VE‐cadherin* increased by shAR in SK and HA22T cells. (B) *shNotch4* inhibits the expression of Notch4 increased by shAR in SK and HA22T cells. (C) *shVE‐cadherin* and *shNotch4* suppresses VM formation increased by shAR in SK and HA22T cells. (D) Quantification of VM formation in SK and HA22T cells in Figure 3C; *P < .05, **P < .01, compared with shAR + PLV + PLV group; ^##^ *P* < .01, compared with PLKO + PLV + PLV group

As AR can suppress the formation of VM in SK and HA22T, and can also suppress the expression of VE‐cadherin and Notch4 in HCC, it is likely that AR may suppress the formation of HCC VM by inhibiting the expression of Notch4 and VE‐cadherin. To directly test this theory, we knocked down *Notch4* and *VE‐cadherin* expression with *shVE‐cadherin* and *shNotch4*, and observed the effects on the HCC VM formation following *shAR*. This blocking experiment revealed that *shVE‐cadherin* and *shNotch4* significantly decreased the expression of *VE‐cadherin* and *Notch4* increased by *shAR* in SK and HA22T cells. Although *shVE‐cadherin* or *shNotch4* alone could partially reverse the formation VM increased by shAR in SK and HA22T cells, combined use of *shVE‐cadherin* and *shNotch4* completely reversed the effects in SK and HA22T cells (Figure 3A,B). These results show that AR suppresses the formation of HCC VM by inhibiting the expression of *Notch4* and *VE‐cadherin* in SK and HA22T cells.

### 3.4 AR increases the expression of *miR‐7‐5p* in SK cells

**FIGURE 4:**
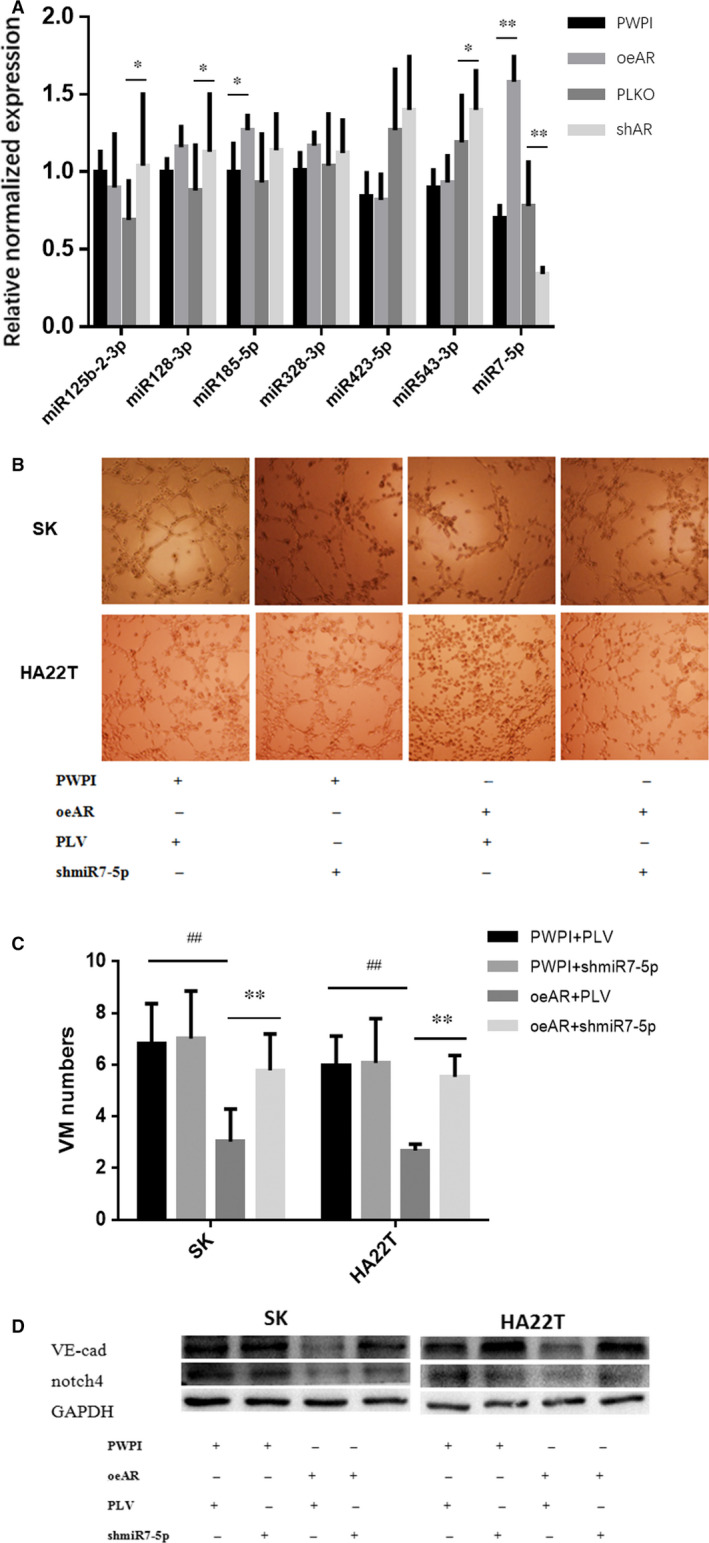
AR increases the expression of *miR‐7‐5p* in SK cells. (A) Relative normalized expression of miRNAs by qRT‐PCR analysis in SK cells. oeAR significantly up‐regulates expression of *miR‐7‐5p* and shAR down‐regulates the expression of *miR‐7‐5p* in SK cells. (B) The effect of *shmiR‐7‐5p* on VM formation. The number of VM formations were counted and compared in SK and HA22T cells. (C) Quantification of VM formation in SK and HA22T cells. (D) The effect of *shmiR‐7‐5p* on expression of *VE‐cadherin* and *Notch4* in SK and HA22T. Western blotting was performed as described in Materials and Methods. ^##^ *P* < .01 compared with PWPI + PLV group. **P < .01 compared with oeAR + PLV group

To determine the molecular mechanisms underlying AR suppression of VM formation in HCC cells through inhibiting the expression of *VE‐cadherin* and *Notch4*, we utilized miRNAs. As miRNAs can target multiple genes, it is possible that a single miRNA can target both *VE‐cadherin* and *Notch4*. Through online databases (TargetScan, miRDB and MicroCosm Targets), we identified a subset of miRNAs that can do so, including *miRNA185‐5p, miRNA328‐3p, miRNA423‐5p, miRNA542‐3p, miRNA7‐5p, miRNA128‐3p and miRNA125‐3p*. Further qRT‐PCR analysis of these miRNA under the impact of AR in SK cells led to the identification of *miRNA‐7‐5p*, which is increased in response to oeAR, while reduced by AR knockdown through shRNA (Figure 4A). To further confirm that AR suppressed the formation of VM via *miR‐7‐5p/VE‐Cadherin/Notch4* signals in HCC, we investigated the effect of *miR‐7‐5p* inhibition on VM formation and expression of *VE‐cadherin* and *Notch4* in SK and HA22T cells. The results revealed that inhibition of *miR‐7‐5p* rescued VM formation blocked by AR (Figure 4B,C) in SK and HA22T cells. *miR‐7‐5p* inhibition also reversed expression of *VE‐cadherin* and *Notch4* that were down‐regulated by AR in SK and HA22T cells (Figure 4D). These results further confirmed that AR suppressed the formation of VM via *miR‐7‐5p/VE‐Cadherin/Notch4* signals in HCC.

### 3.5 *miR‐7‐5p* regulation of *VE‐cadherin* and *Notch4* expression

**FIGURE 5:**
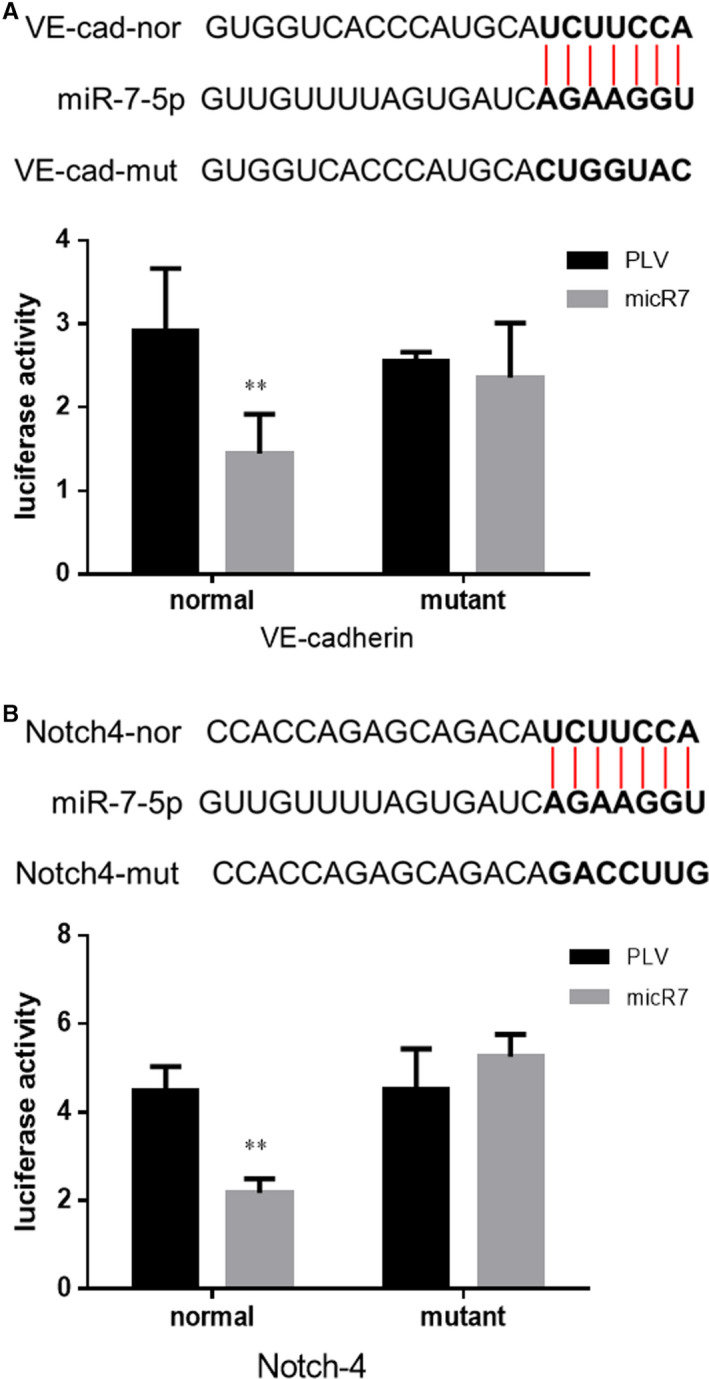
AR inhibits the expression of the *VE‐cadherin* and *Notch4* in SK cells. (A) Luciferase activity of miR‐7‐5p binding to the 3′UTR of *VE‐cadherin* in SK cells. (B) Luciferase activity of miR‐7‐5p binding the 3′UTR of *Notch4* in SK cells. **P < .01 compared with PLV group

To further dissect the mechanisms by which *miR‐7‐5p* decreased *VE‐cadherin* and *Notch4* expression at a molecular level, we used the luciferase reporter assay to determine whether *miR‐7‐5p* directly targeted the 3′UTR of *VE‐cadherin* and *Notch4* mRNA to suppress their expression. We first identified potential miRNA responsive elements that matched the seed sequence of *miR‐7‐5p* in the 3′UTR of the *VE‐cadherin* and *Notch4* genes. We then mutated this miRNA‐responsive element and cloned both wild‐type and mutated miRNA responsive elements into the Renilla luciferase vector. Luciferase assay results revealed that *miR‐7‐5p* decreased the levels of *VE‐cadherin* and *Notch4* containing the wild‐type 3′UTR, but not of the mutated constructs (Figure 5A,B), suggesting *miR‐7‐5p* might directly target the 3′UTR of *VE‐cadherin* and *Notch4* to suppress their expression.

### 3.6 AR inhibits the expression of *circRNA7*

**FIGURE 6:**
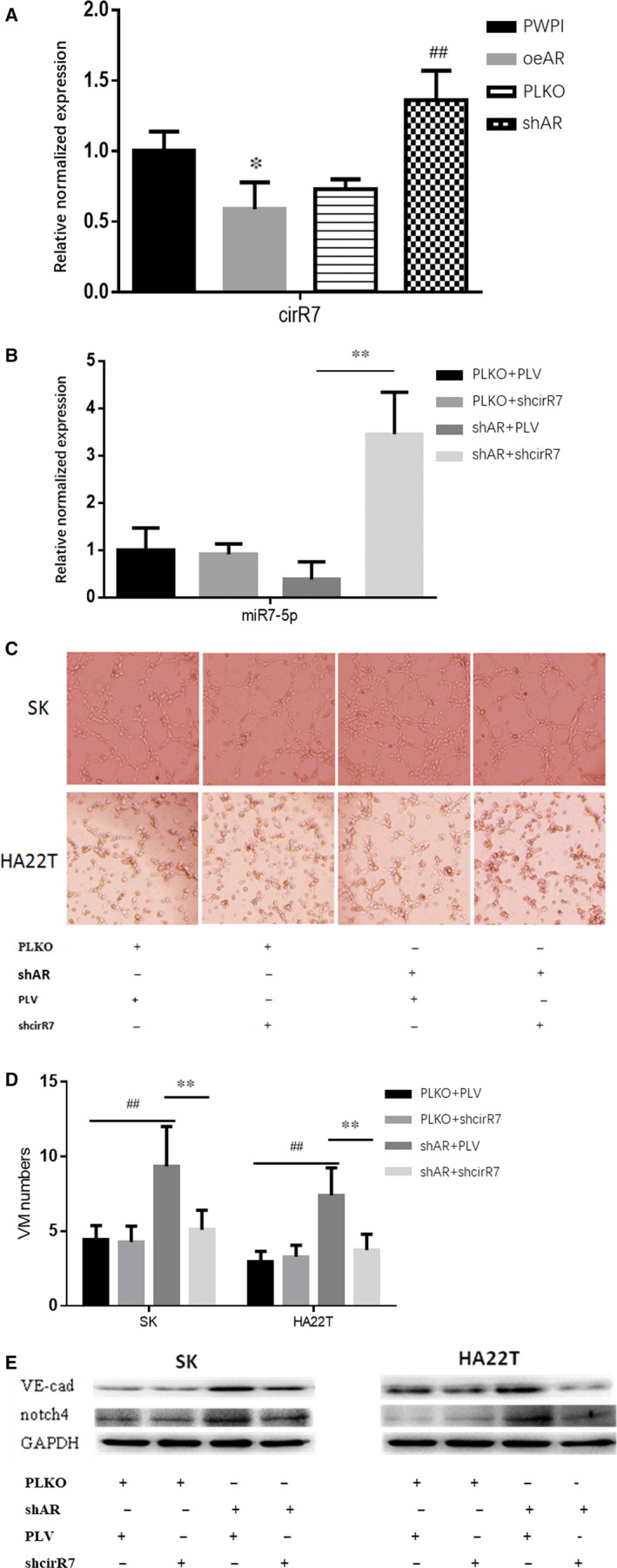
AR inhibits the expression of *circRNA7* in SK cells. (A) Relative normalized expression of *circRNA7* by qRT‐PCR analysis in SK cells. oeAR significantly suppressed expression of *circRNA7* and shAR increased expression of *circRNA7* in SK cells. (B) The effect of *shcircRNA7* on gene expression of *miR‐7‐5p* in SK cells. (C) The effect of *shcircRNA7* on VM formation in SK and HA22T cells. (D) Quantification of VM formation. (E) The effect of *shcircRNA7* on expression of *VE‐cadherin* and *Notch4* in SK and HA22T cells. ^##^ *P* < .01 compared with PLKO + PLV group. **P < .01 compared with shAR + PLV group

To determine the molecular mechanism responsible for how AR regulated *miR‐7‐5p* expression in HCC cells, we focused on the potential role of *circRNA7*, which has been reported to be able to sponge over 50 *miRNA‐7‐5p* in its sequence. We found that oeAR could significantly suppress the expression of *circRNA7*, while *shAR* had the opposite effect in SK cells (Figure 6A). The results suggested that AR increased the expression of the *miR‐7‐5p* through inhibiting *circRNA7* expression in HCC cells. To further confirm that AR suppressed the formation of VM via *circRNA7/miR‐7‐5p/VE‐cadherin/Notch4* signals in HCC, we investigated the effect of *shcircRNA7* on VM formation and expression of *miR‐7‐5p, VE‐cadherin*, and *Notch4* in HCC cell lines. The results revealed that *shcircRNA7* increased the expression of *miR‐7‐5p* that had been decreased by *shAR* (Figure 6B) and decreased formation of VM that had been increased by *shAR* (Figure 6C,D). It also reversed the expression of *VE‐cadherin* and *Notch4* that had been up‐regulated by *shAR* in SK and HA22T cells (Figure 6E). Thus, these results suggest that AR may regulate *circRNA7* expression to impact *miRNA‐7‐5p* expression as well as its downstream effect on *VE‐cadherin* and *Notch4* expression and VM formation.

### 3.7 AR regulates the expression of *circRNA7* in HCC cells

**FIGURE 7:**
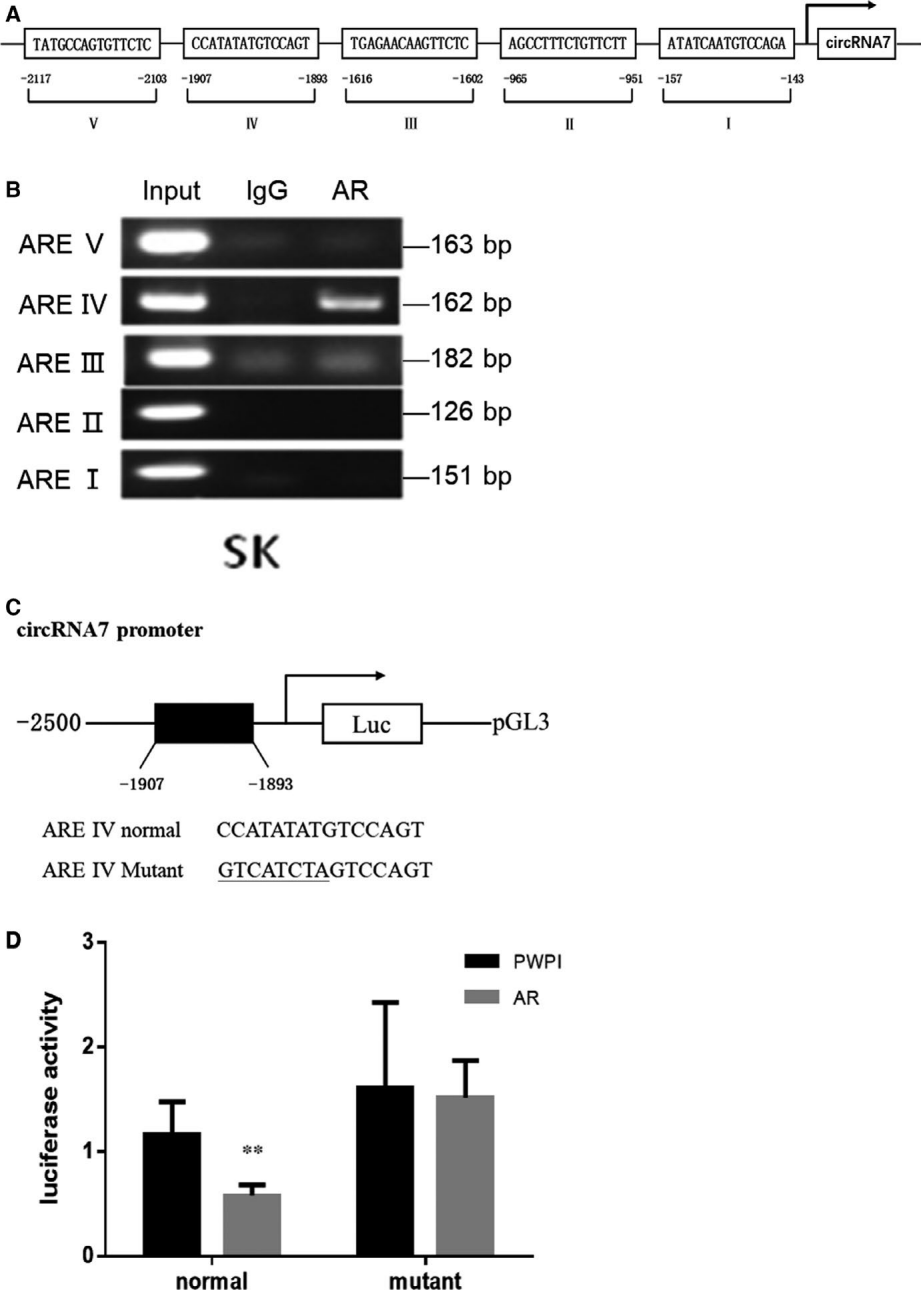
AR regulates the expression of *circRNA7* in SK cells. (A) Online software (JASPAR and PROMO) was used to predict the binding sites of AR and *circRNA7* promoter. (B) ChIP assay for AR directly targeting the *circRNA7* promoter in SK cells. (C) The AR‐responsive element was mutated. (D) Luciferase activity of AR directly targeting the *circRNA7* promoter in SK. **P < .01 compared with PWPI group

To further dissect the mechanism by which AR decreases *circRNA7* expression at a molecular level, we used the luciferase reporter assay to determine whether AR directly targeted the *circRNA7* host gene promoter to suppress its expression. We first identified five potential AR targets in the promoter (Figure 7A) and found one potential AR responsive element that matched the seed sequence of AR in the promoter of the *circRNA7* host gene by ChIP assay (Figure 7B). We then mutated this AR responsive element and cloned both the wild‐type and mutated AR responsive element into a pGL3 reporter construct (Figure 7C). The results revealed that AR could decrease the expression of *circRNA7* containing the wild‐type host gene promoter, but not the mutated constructs (Figure 7D), suggesting that AR might directly target the *circRNA7* host gene promoter to suppress its expression.

## 4 DISCUSSION

HCC, a primary neoplasm of the liver, is a common primary cancer with multifaceted molecular pathogenesis pathways. HCC has become the fourth most prevalent malignant tumour worldwide, and the fourth leading cause of cancer‐related deaths. Most importantly, the incidence of HCC is increasing.^1^, ^2^ Invasion and metastasis are two fundamental properties which determine the prognosis of HCC patients.^4^, ^5^ Currently, there are no available effective treatments. Therefore, understanding the mechanisms that contribute to HCC progression is an urgent issue for enhancing patient survival.

Recent studies indicated that targeting AR could suppress HBV‐, HCV‐ and carcinogen‐induced HCC development at early stages. Several clinical trials based on these discoveries have been proposed to test their efficacy to suppress HCC progression.^26^, ^27^, ^28^ However, clinical trials using various anti‐androgens for treating HCC, including leuprorelin, flutamide, ketoconazole, tamoxifen and cyproterone, yielded inconsistent results.^29^, ^30^, ^31^, ^32^, ^33^ Recently, Ma et al demonstrated the dual roles of AR in HCC, indicating that AR might function as a metastasis suppressor at advanced stages. This implies that targeting AR could be stage dependent.^10^ However, the molecular mechanisms by which AR signals suppress HCC late‐stage progression remain unclear.

VM, a new pattern by which tumour tissues nourish themselves, is a means for supplying blood to tumours. VM is also involved in metastasis and invasion, and is implicated in poor prognosis in HCC.^21^ Our laboratory found that AR can influence VM formations in RCC. However, whether AR can influence VM formation in HCC was still unclear. Therefore, we investigated the effect of AR on the formation of VM in HCC. The results revealed that AR can significantly inhibit the formation of HCC VM in vitro, suggesting that AR may inhibit HCC metastasis progression by blocking VM formation. In addition, oeAR down‐regulated gene expression of *Notch4* and *VE‐cadherin* in SK and HA22T cells, and *shAR* increased protein expression in both SK and HA22T cells. These results suggest that AR can suppress the formation of HCC VM by inhibiting the expression of *Notch4* and *VE‐cadherin*. To further confirm these results, we knocked down *Notch4* and *VE‐cadherin* and observed the effects of *shAR* on the formation of HCC VM. Results revealed *shVE‐cadherin* or *shNotch4* alone could partially reverse the formation of HCC VM that had been increased by *shAR shVE‐cadherin* and *shNotch4* together completely reversed the formation of HCC VM that had been increased by *shAR*. These results confirmed that AR suppressed the formation of HCC VM via inhibiting the expression of *Notch4* and *VE‐cadherin*.

miRNAs are a class of endogenous non‐coding RNAs that are approximately 22 nucleotides long and can bind target specific bases of mRNA, leading to their degradation or inhibition of mRNA translation.^34^ Circular RNAs (circRNAs) are another class of non‐coding RNAs that interact with miRNAs through miRNA response elements. Thus, they can act as competitive endogenous RNAs through binding and sponging of miRNAs, thereby blocking the inhibition of miRNAs to up‐regulate the expression of target genes.^35^ Indeed, we identified that this relationship is likely the molecular mechanism mediating the effect of AR on HCC VM formation. We found AR down‐regulated the expression of *circRNA7*, up‐regulated the expression of *miR‐7‐5p* and suppressed VM formation in HCC *ShcircR7* blocked VM formation and the expression of *VE‐cadherin* and *Notch4* that had been increased by *shAR* in HCC, and *shmiR‐7‐5p* rescued VM formation and expression of *VE‐cadherin* and *Notch4* that had been decreased by oeAR in HCC. Altogether, these results indicate that AR suppresses the formation of VM via *circRNA7/miRNA7‐5p/VE‐Cadherin/Notch4* signals in HCC.

To further dissect the mechanism by which *miRNA‐7‐5p* decreased *VE‐cadherin* and *Notch4* at a molecular level, we performed luciferase reporter assays to determine the binding of *miRNA‐7‐5p* to the 3′UTR of the *VE‐cadherin* and *Notch4* mRNA. The results revealed that *miRNA‐7‐5p* directly targets the 3′UTR of *VE‐cadherin* and *Notch4* to suppress their expression, and that AR can directly target the promoter of *circRNA7* to suppress its expression in HCC. In conclusion, AR can inhibit the formation of HCC VM via down‐regulation of *AR‐ circRNA7/miRNA7‐5p/VE‐Cadherin/Notch4* signals, and these mechanisms may contribute to develop new strategies towards defeating HCC.

## CONFLICT OF INTEREST

The authors confirm that there are no conflicts of interest.

## AUTHOR CONTRIBUTION

**Shixiang Bao:** Conceptualization (equal); Methodology (lead); Project administration (equal); Writing‐original draft (equal). **Shuai Jin:** Data curation (lead); Methodology (equal); Software (lead). **Chunhua Wang:** Methodology (equal); Writing‐original draft (equal). **Peipei Tu:** Data curation (supporting); Methodology (equal). **Kongwang Hu:** Conceptualization (equal); Writing‐review & editing (equal). **Jingtao Lu:** Conceptualization (equal); Project administration (lead); Resources (lead); Supervision (lead); Writing‐review & editing (equal).

## ETHICAL APPROVAL

This study does not contain any experiments with human participants or animals performed by any of the authors.

## Supporting information

Table S1

## Data Availability

All data generated or analysed during this study are available from the corresponding author upon request.
