# Supplementary figures and images for: Androgen receptor suppresses vasculogenic mimicry in hepatocellular carcinoma via circRNA7/miRNA7‐5p/VE‐cadherin/Notch4 signalling
Source: J Cell Mol Med. 2020 Oct 28;24(23):14110–20. doi: 10.1111/jcmm.16022 (PMC7754040; doi:10.1111/jcmm.16022)

**Supplemental Table**

primer sequences for ChIP assay


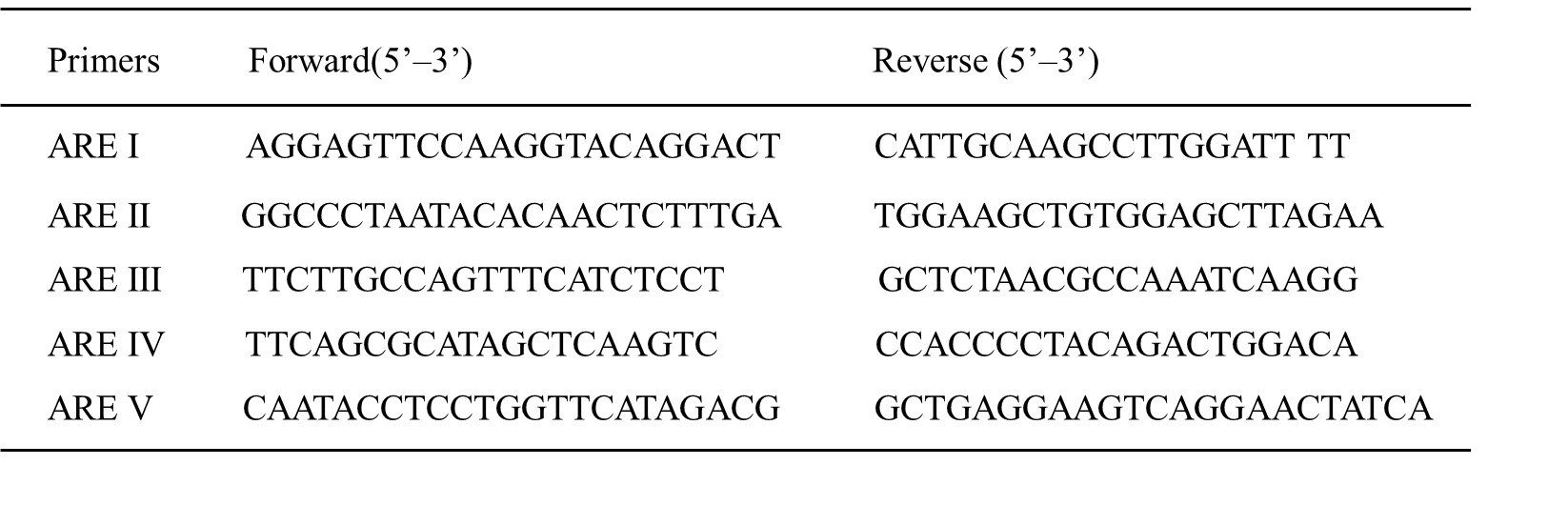

Supplement: Supplementary file 1 — Table S1 [file JCMM-24-14110-s001.docx]
